# The Burden of Concomitant Spinal Injury in the Setting of Traumatic Brain Injury that Required Admission to ICU—Lessons from a Tertiary Neurosurgery Center

**DOI:** 10.1007/s12028-026-02454-x

**Published:** 2026-02-18

**Authors:** Andreas K. Demetriades, Imran Shah, Ali R. Syed, Charles Wallis, Wilco Peul

**Affiliations:** 1 Edinburgh Spinal Surgery Outcome Studies Group, Department of Neurosurgery, Royal Infirmary Edinburgh, NHS Lothian, Edinburgh, Scotland, UK; 2 Department of Neurosurgery, Leiden University Medical Centre, Leiden, The Netherlands; 3 Department of Clinical Radiology, Royal Infirmary Edinburgh, NHS Lothian, Edinburgh, Scotland, UK; 4 Department of Intensive Care, Western General Hospital, NHS Lothian, Edinburgh, Scotland, UK; 5 Department of Neurosurgery, Haaglanden Medical Center, The Hague, The Netherlands

**Keywords:** Traumatic brain injury (TBI), Spinal trauma, Spinal cord injury, Vertebral fracture, Concomitant craniospinal trauma, Neurotrauma, Polytrauma

## Abstract

**Introduction:**

While individually both traumatic brain injury (TBI) and traumatic spinal injury have been studied extensively, the relationship between concurrent TBI and spinal column and/or cord injuries has not. We aimed to identify basic epidemiology, patterns of injury, and patient outcomes from a population served by a tertiary neurosurgery center.

**Methodology:**

A database was built of patient data on admissions to an adult intensive care unit with a TBI over a 12-year period. Electronic patient records, sourced from the database of the Scottish Intensive Care Society Audit Group (SICSAG), were analyzed retrospectively to identify patients who had suffered both a TBI and a concomitant spinal column/cord injury. Data were analyzed on demographics, mechanism of injury, neurological parameters on arrival, clinical management, discharge destinations, and patient outcomes.

**Results:**

Out of 560 patients admitted to ICU with TBI, 85 (85/560; 15.2%) were found to have concomitant spinal injuries. Concomitant thoracolumbar spinal injuries (34/85) were more common than cervical spine injuries (30/85), with 21 patients sustaining both cervical and thoracolumbar injuries.

Among the concomitant brain and spinal trauma, spinal cord injuries (SCI) were identified in 16/85 patients (16/560; 2.9%). Outcome assessment revealed 18/85 mortality during index admission, while 36/85 patients required further neurorehabilitation. Concomitant spinal injury was associated with more severe TBI, with 60/85 patients having GCS ≤ 8, and poorer outcomes, with 20% of patients dying during admission.

Dichotomizing between cervical and thoracolumbar regions, more SCIs occurred in cervical (10/85) than thoracolumbar (6/85) trauma. SCIs were more pronounced if GCS ≤ 8.

**Conclusions:**

Among TBI requiring ICU admission, there were 15.2% concomitant spinal column injuries, including 2.9% SCI. Lessons on the patterns of concomitant craniospinal injury and their outcomes can help stratify resources, improve the assessment and diagnosis of such complex trauma, and guide future protocols to improve patient outcomes.

**Supplementary Information:**

The online version contains supplementary material available at 10.1007/s12028-026-02454-x.

## Introduction

Traumatic brain injury (TBI) can be defined as an acquired injury to the brain due to an external mechanical force causing a “bump, blow, or jolt” to the head, or a penetrating injury that causes a disruption to the normal function of the brain [1]. Meanwhile, traumatic spinal injuries (TSI) are defined as a multitude of injuries affecting the bony and/or ligamentous structures of the spinal column, and/or injuries affecting the spinal cord [2]. Spinal cord injuries (SCI) in this setting can occur due to sudden trauma to the spine, resulting in possible spinal ligament tearing as well as bone fragmentation from the spinal column causing cord compression. These primary injuries that occur to both brain and/or spinal cord would then trigger a secondary injury process that causes further chemical and mechanical damage to the brain and spinal cord tissues, resulting in neurological dysfunction [3, 4].

Due to the close anatomical association between the head and spine, injury to either one of these structures increases the chance of a combined craniospinal injury, particularly in a traumatic setting [5]. This can be attributed to several mechanisms of injury, such as the impact and orientation of forces applied to the head, for which the transmitted forces cause cervical spine loading and buckling [6]. Thus, in any situation involving an injury to either structure, it is important to investigate and rule out any combined injury due to the potential neurological and life-threatening consequences of missing such injuries [7]. The Global Burden of Disease study in 2016 has shown that both traumatic brain and spinal injuries continue to increase in prevalence globally and carry a risk of neurological deficits, which remain a significant cause of mortality and morbidity [8]. There have been several studies on the incidence of isolated TBI or TSI; about 50–60 million people worldwide are affected by TBI each year; and a meta-analysis has shown that the overall global incidence of a traumatic spinal injury was 10.5 cases per 100,000 people annually [9, 10].

However, the inter-relationships between TBI and concomitant spinal column and/or cord injuries have not been studied extensively, with only few specific studies available. A meta-analysis has shown that the prevalence of concomitant cervical spine injury in adult patients with TBI was 6.5%. Additionally, a cohort study of patients in a level 1 trauma center in Verona, Italy, showed that the rate of concomitant upper cervical spine injury among patients admitted with TBI was 1.4% [11, 12].

Current evidence highlights several gaps. First, there is limited high-quality prospective data on optimal screening protocols, especially in obtunded or polytrauma patients, and controversy remains regarding the best imaging modalities and timing for clearance of the cervical spine [13, 14]. Second, outcome data are heterogeneous; while some studies show that concomitant injuries do not universally worsen mortality or functional outcomes, others report that patients with both TBI and spinal cord injury have higher rates of rehospitalization, reduced functional recovery, and increased pain and depression at 1 year [15–17]. Third, there is a lack of consensus on the timing and prioritization of surgical interventions in patients with both TBI and spinal cord injury, with evidence that concomitant TBI may delay time-sensitive spinal surgery [14, 17].

The aim of this study was to identify, among a cohort of patients who suffered a TBI, any basic epidemiological patterns of concomitant spinal column and cord injury, within the setting of a population served by the tertiary neurosurgical center of Southeast Scotland. We believe that developing a better understanding of epidemiological patterns of these concomitant injuries would aid in developing more effective screening protocols, guide specialized management of dual neurotrauma injuries, and ultimately improve patient outcomes.

## Hypotheses

Primary outcome: length of stay (LOS). We hypothesized that among adults with concomitant craniospinal trauma, cervical spinal column injury is associated with longer hospital LOS than non-cervical injury (thoracolumbar or both regions).

Secondary outcome: discharge destination. Because cervical injuries more often produce neurological deficits, respiratory/airway issues, and mobility limitations requiring specialist inpatient rehabilitation, patients with cervical injury will be less likely to be discharged home and more likely to go to rehabilitation or transfer than those with non-cervical injury.

## Methods

This study was a single-center retrospective cohort design, capturing patients that were admitted to the intensive care unit (ICU) at the Western General Hospital, Edinburgh. This site, once the headquarters of the Department of Clinical Neurosciences (DCN), was the tertiary neurosurgical center prior to moving headquarters to the Royal Infirmary, Edinburgh, in 2020. The DCN represents the regional neurosurgery referral center for the southeast region of Scotland, accepting acute patients from eight or more other hospitals, serving a region of approximately 1.5 million [18].

## Definitions

For the purpose of this study, TBI was defined as a head injury resulting from trauma that caused either a reduced Glasgow coma scale (GCS) score on arrival, with or without an abnormal head imaging result on computed tomography (CT) or magnetic resonance imaging (MRI). Abnormal imaging results included findings of extradural hemorrhage, subdural hemorrhage, subarachnoid hemorrhage, parenchymal hemorrhage, intraventricular hemorrhage, brain contusion, skull fracture, or a combination of these. We defined patients with a spinal column injury as patients sustaining a ligamentous injury and/or fracture in either the cervical spine; thoracolumbar spine; or a combination of these, as detected on CT and/or MRI imaging. We defined patients as having severe TBI (s-TBI) when GCS was 8 or less on arrival.

## Database

Information regarding adult patients admitted with TBI was obtained via the Scottish Intensive Care Society Audit Group (SICSAG), which maintains a national database of all patients admitted to adult general ICU departments in Scotland since 1995 [19]. A new dataset specific to this study was created comprising all patients admitted to the ICU department, Western General Hospital, Edinburgh, during the 12-year period between 1 September 2008 and 1 September 2019. Further granularity was added by cross-referencing and further data acquisition using the local electronic patient record system used in NHS Lothian, TrakCare (Intersystems) [20].

## Inclusion Criteria

Patients over 16 years of age, the legal age when a child becomes an adult observed in NHS Scotland, who had suffered TBI, who had been admitted to the Intensive Care Unit (ICU), and who had also suffered any concomitant spinal trauma, including spinal column and spinal cord injuries, were included.

## Exclusion Criteria

Any patients with incomplete datasets were not included. Patients with no concomitant spinal injury were excluded. In Scotland, patients with a primary spinal cord injury are primarily admitted to the Queen Elizabeth National Spinal Injuries Unit in Glasgow [21], but not if they have any coma-inducing head injury and/or the need for ICU support for TBI, thus our dataset only includes patients from our tertiary region who had cranial and spinal injury and who needed ICU admission due to the severity of their TBI. Specifically, any patients with SCI with only mild-to-moderate TBI who were not admitted to our ICU were therefore not included in this study.

## Data Parameters

Demographics, mechanism of injury, radiological parameters, and clinical parameters were collected, including the presence of concomitant spinal column and/or cord injury, GCS score on arrival, type of clinical management, discharge destination, and patient outcomes at 1 year.

## Statistical Analysis

Descriptive analyses summarized baseline demographics, injury patterns, and outcomes. Continuous data are reported as median [IQR] (with mean ± SD provided where helpful), and categorical data as *n* (%). For categorical predictors we used Pearson’s chi-squared test; when any expected cell count was < 5, we used two-sided Fisher’s exact test and reported unadjusted odds ratios (ORs) with exact 95% CIs. For two-group comparisons of continuous outcomes, we used the Mann–Whitney *U* test and expressed the effect as the Hodges–Lehmann (HL) median difference with 95% CI. For comparisons across ≥ 3 groups we used the Kruskal–Wallis test (a significant result indicates that at least one group median differs); when appropriate, pairwise Mann–Whitney tests with Holm adjustment and HL differences were examined.

Two clinically relevant binary variables were created: prolonged length of stay (LOS) defined as ≥ 30 days (vs. < 30), and favorable discharge defined as home (vs. not home: rehabilitation or hospital transfer).

We performed univariate analyses and report for each comparison the effect estimate (OR where applicable), its 95% CI, and the corresponding *p*-value.

In terms of multivariable analyses, prolonged LOS was modelled with Firth’s penalized logistic regression to reduce small-sample and separation bias. The prespecified model included age per 10 years, severe GCS (≤ 8 vs. > 8), and spinal region (cervical vs. non-cervical), chosen a priori to respect the events-per-variable (EPV) constraint. Results are presented as adjusted ORs with profile-likelihood 95% CIs. For discharge destination, the number of “home” events was small and several predictors produced sparse/zero cells, yielding insufficient events per variable for a stable multivariable model. Accordingly, we report unadjusted exact analyses for discharge destination and interpret them accordingly.

All tests were two-sided with statistical significance at < 0.05. Analyses were performed in Stata BE 18.5 (StataCorp, College Station, TX); figures were prepared in GraphPad Prism.

## Results

**Fig. 1:**
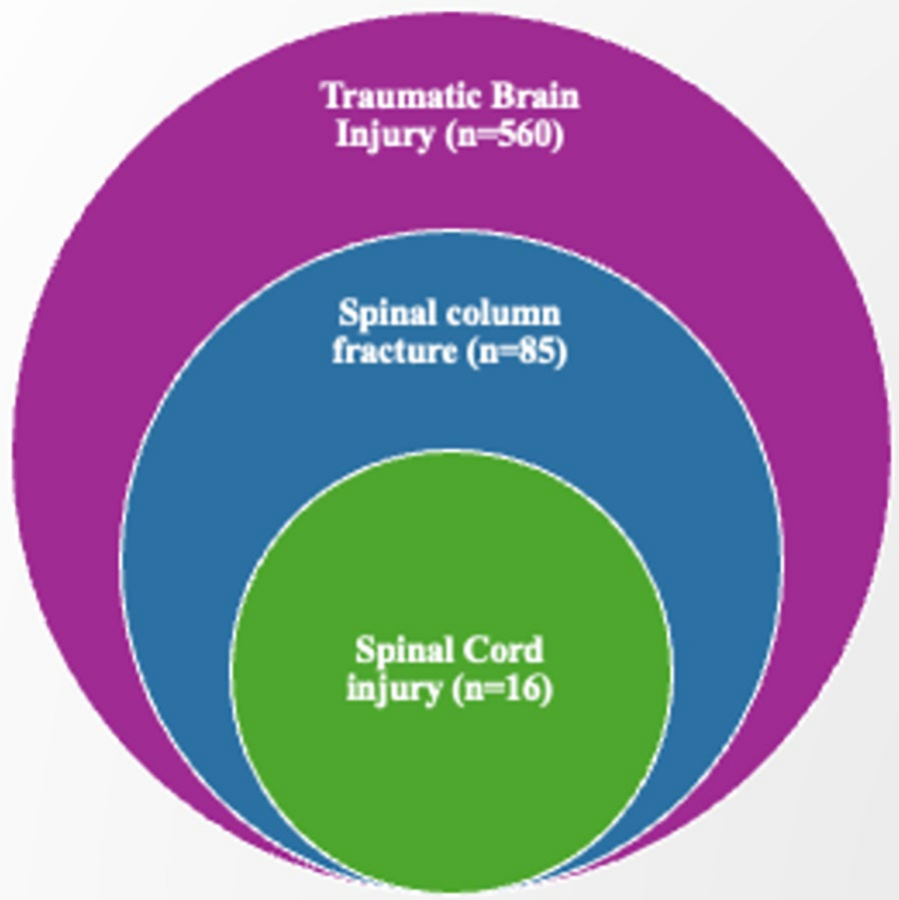
Total number of TBIs, spinal column fractures, and spinal cord injuries

Within the 12-year period studied, a total of 560 patients were identified who had suffered TBI that required admission to our ICU. Of these, 441/560 (79%) were male. Out of these 560 patients, 85 patients (85/560, 15.2%) also suffered concomitant spinal column injuries alongside the TBI, which was confirmed on spinal imaging. Out of these 85 patients with concomitant spinal column injuries and TBI, we identified 16 patients (16/560, 2.9%) who had sustained a spinal cord injury (Fig. 1).

## Characteristics of Patients with TBI with Concomitant Spinal Column Injuries

Table 1 presents several characteristics of these 85 patients. While 30 of these patients had also suffered a cervical spine injury, 34 had a concurrent thoracolumbar spine injury. Another 21 patients with TBI had both cervical and thoracolumbar concomitant injuries. The mean age of these 85 patients was 42 years (range 17–77; median 44) years. Within the concomitant craniospinal trauma cohort, 73/85 patients (85.9%) were male, while the remaining 12/85 were female (14.1%). The ratio of male and female individuals with TBI who suffered concomitant spinal injury among the whole group were 73/441 (17%) male and 12/119 (10%) female.

**Table 1:** Patients with TBI and concomitant spinal column injury (*n* = 85)

Spinal column region affected	Cervical	30 (35.3%)
	Thoracolumbar	34 (40.0%)
	Both	21 (24.7%)
Mechanism of injury	Motor vehicle accidents (MVA)	38 (44.7%)
	High-energy fall (HEF)	35 (41.2%)
	Low-energy impact	5 (5.9%)
	Bicycle	6 (7.1%)
	Assault	1 (1.1%)
Gender	Male	73 (85.9%)
	Female	12 (14.1%)
Age (years)	40 and younger	38 (44.7%)
	41–65	40 (47.1%)
	Older than 65	7 (8.2%)
Management	Medical	48 (56.5%)
	Surgical	37 (43.5%)
GCS score on assessment	13–15	16 (18.8%)
	9–12	9 (10.6%)
	8 and less	60 (70.6%)
Discharge destinations	Home	14 (16.5%)
	Neurorehabilitation center	36 (42.3%)
	Transferred to other hospitals	17 (20.0%)
	N/A (Patients died during admission)	18 (21.2%)
Patient outcomes	Alive at 1 year	63 (74.1%)
	Readmission	2 (2.4%)
	Died (during admission and at 1 year)	20 (23.5%)

Several mechanisms of injury that resulted in concomitant injuries were identified. Most patients were admitted due to motor vehicle accidents (MVA) (38 patients) and high-energy falls (HEF) (35 patients); these two predominant mechanisms of injury together accounted for 86.1% of injuries. The remainder were due to low-energy impact (5 patients), bicycle accidents (6 patients), and there was one isolated case of an assault.

Just over half of the patients who had sustained a concomitant spinal column injury were managed medically (48 patients), while the rest were managed surgically. The type of medical management ranged from spinal bracing and collars for stabilization to intracranial pressure (ICP) monitoring while intubated in the ICU. Surgical management of concomitant brain and spinal injuries involved procedures such as craniectomies, craniotomies, burr holes, washout and closure of head wounds, and spinal instrumented stabilizations. Most patients in the concomitant craniospinal trauma group had a GCS of 8 or less (70.6%), with 10.6% having a GCS score of between 9 and 12, and 18.8% a GCS score of 13–15. This latter group comprised patients who were deemed at high risk of neurological deterioration and admitted for close monitoring, such as with extradural or subdural hematoma. After a short period in the ICU, such patients with a GCS of 13–15 were deemed well enough to be stepped down onto the neurosurgical ward.

The discharge destination for patients was assessed, and 36/85 patients (42.3%) were discharged to a neurorehabilitation center, specifically the Astley Ainslie Hospital, which is the base for adult neurorehabilitation after a neurological injury in Edinburgh [17]. A fifth (17/85 patients, 20%) were transferred to other hospitals outside of Edinburgh after recovering from the acute phase of their injury and no longer requiring any ICU intervention. A sixth (14/85 patients, 16.5%) made a substantial recovery and was deemed medically fit to be discharged home without requiring any inpatient neurorehabilitation. The remaining 18 patients (21.2%) died during their admission.

Looking into the cause of deaths for these 18 patients specifically, 10 patients had withdrawal of life-sustaining therapy in the ICU, as it was deemed that their TBI was severe with a poor prognosis. Four patients were diagnosed with brainstem death, two patients had inpatient cardiac arrests, one developed sepsis leading to multiorgan failure, and the last patient developed hospital-acquired-pneumonia and subsequently died from it.

Outcomes at 1 year after injury showed that 63/85 patients (74.1%) were alive, including 2/85 (2.4%) who needed readmission within that period. Two patients were readmitted with ventriculomegaly, but only one was deemed to have clinical hydrocephalus, and underwent a ventriculoperitoneal shunt as well as a cranioplasty 6 months after injury.

At 1 year post-injury, mortality within the concomitant craniospinal injury group was 23.5% (20/85). In addition to 18 patients who had died during their primary admission, two further patients died within 1 year of their injury; one patient died from liver decompensation, while the other patient died after being transferred to another hospital outside Edinburgh. The cause of death for this patient is unknown.

## Spinal Column Injuries

**Fig. 2:**
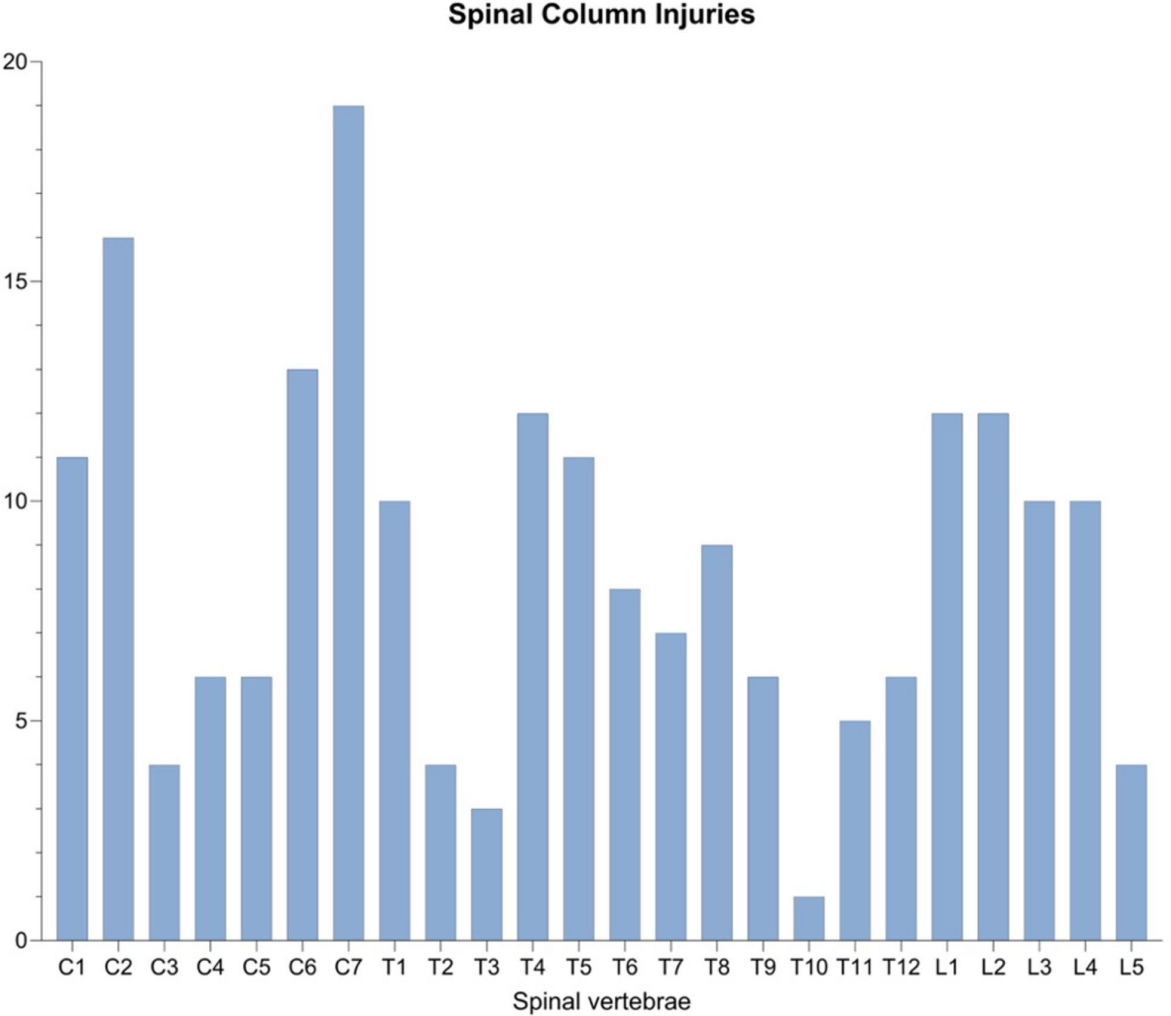
Anatomical distribution of concomitant spinal column injuries in those admitted to ICU with a TBI

A large variation was observed in regard to the pattern of spinal column injury, and this involved any vertebrae from C1 to L5 (Fig. 2.). Although thoracolumbar fractures predominated (41.8%), the most common vertebrae that were injured were C7 (19 patients) and C2 (16 patients). The highest number of thoracolumbar injuries were identified at T4, L1, and L2, with 12 patients sustaining an injury involving each of these vertebrae.

## Features of Patients with Concomitant Cervical Spinal Injury

We found that 51/85 patients (60.0%) suffered concomitant cervical spine injuries, including those both isolated and in combination with thoracolumbar injuries. The features of these patients can be seen in Table 2. A significant majority of these patients had a reduced GCS on initial assessment, with 36/50 patients (70.6%) having a GCS of 8 or less. About half of these patients (25/51) with concomitant cervical spine injuries were admitted due to MVA, while 33.3% (17/50 patients) were admitted due to HEF. There was an equal number of patients admitted due to bicycle accidents or low-energy impact with four patients each (total of 15.6%). Only one patient was admitted due to an assault. Despite 70.6% of these patients having a GCS of 8 and lower, indicating severe TBI, more than half of these patients were managed medically (62.7%), while the remaining 37.3% were managed with surgical interventions. Lastly, we found that 19.6% (10/51) of patients with a concomitant cervical spine injury had also suffered a cervical spinal cord injury alongside this. Each of these patients had suffered a cervical cord injury that was adjacent to the segment of the cervical spine injured.

**Table 2:** Patients with concomitant cervical spine injuries (*n* = 51)

GCS 8 and less	36 (70.6%)	
Mechanism of injury	High-energy fall	17 (33.3%)
	Motor vehicle accidents	25 (49.1%)
	Bicycle	4 (7.8%)
	Low energy	4 (7.8%)
	Assault	1 (2.0%)
Spinal cord injury	10 (19.6%)	
Management	Medical	32 (62.7%)
	Surgical	19 (37.3%)

## Associations of Patients with a Severe TBI (GCS Score of 8 or Less)

The majority of patients identified with a concomitant spinal column injury were found to have severe TBI, with 60/85 patients (70.6%) having a GCS of 8 and less on initial assessment upon arrival to the emergency department. Table 3 presents characteristics and associations of patients with severe TBI. The distribution of the mechanism of injury surrounding these patients was similar to those who had a concomitant cervical spine injury. In order of incidence, just over half of these 60 patients (51.7%) had suffered MVA, followed by those suffering HEF with 23/60 patients (38.3%). The remaining patients had been admitted due to a bicycle accident (5.0%), low-energy impact (3.3%), and an assault (1.7%). With regard to how these patients were managed, more than half of patients were managed medically (37/60), and the remaining managed surgically (23/60). Regarding patient discharge destinations, 27/60 patients (45.0%) had been discharged to the local neurorehabilitation center (Astley Ainsley Hospital), 12/60 patients (20.0%) to another hospital for further supportive management once they had recovered from their acute injury, and 6/60 patients (10.0%) were deemed medically well enough to be discharged home. We found that 25% of these patients with severe TBIs died during their admission. We also found that 12 patients with severe TBI were also found to have a concurrent spinal cord injury. There were more patients with cervical cord injuries (8/60 patients) than there were patients with thoracolumbar cord injuries (4/60 patients).

**Table 3:** Patients with a GCS of 8 and less (*n* = 60)

Impact category	HEF	23 (38.3%)
	MVA	31 (51.7%)
	Low	2 (3.3%)
	Bike	3 (5.0%)
	Assault	1 (1.7%)
Management	Medical	37 (61.7%)
	Surgical	23 (38.3%)
Spinal cord injury	Cervical	8 (13.3%)
	Thoracolumbar	4 (6.7%)
Discharge destination	Neurorehabilitation	27 (45.0%)
	Transferred to another hospital	12 (20.0%)
	Home	6 (10.0%)
	N/A (died during admission)	15 (25.0%)

## Associations of Patients with Spinal Cord Injury (SCI)

Out of the 85 patients with concomitant spinal column trauma, 16 patients had an associated spinal cord injury (SCI). The ASIA Impairment Scale for this group comprised one A, eight B, one C, four D, and no E patients, while information for two was not available.

When dichotomizing between cervical and thoracolumbar regions, more SCI occurred with concomitant cervical spinal cord injuries, with 10/85 patients (11.7%). These SCIs were identified on MRI spine, which showed findings such as myelopathic cord changes, cord contusions, cord impingements, high cord signals, hemi-cord injuries, and in one incident, cord transection. This relationship of associated SCI was more pronounced in cases where the GCS was 8 or less; as seen in the previous section, 12/16 patients with a spinal cord injury also had severe TBI.

None of these patients were discharged home. The destination of discharge for these patients was as follows: 6/16 patients were transferred to other hospitals for ongoing management and rehabilitation once their acute injury had been treated, and 5/16 were transferred to neurorehabilitation centers. The remaining 5/16 patients died during initial admission.

## Patient Outcomes at 1 Year

A total of 36 out of 85 patients had been discharged to neurorehabilitation centers for further management of their initial injury. At 1 year post-injury, the number of patients who remained under the care of neurorehabilitation was 30/36, either as an outpatient or inpatient. Out of these 30 patients, 27 had been discharged as an inpatient, but had been routinely followed up as an outpatient in the neurorehabilitation clinics to monitor their neurological function. The other three patients remained as inpatients, each requiring an extensive period of rehabilitation and care tailored to their needs after having sustained severe injuries. The remaining six patients had been discharged without any further follow-up, as their neurological status and function was deemed well enough at the time of discharge.

The 1-year outcomes of the five patients with spinal cord injury who were specifically discharged into neurorehabilitation centers were as follows: one patient’s ASIA grade improved from D at the time of injury to E; two patients’ ASIA grade remained unchanged at B and D, respectively; one improved from B to D; and one was transferred to England and outcome remains unknown.

Of the 6/16 patients who had been transferred to other non-neurorehabilitation hospitals, two died 4 years after injury, one improved from ASIA C to D, and another improved from ASIA B to C, while two others had no change and remained paraplegic at ASIA B.

## Univariate Analyses

None of the baseline categorical variables (gender, mechanism of injury, spinal column level, or GCS severity) differed significantly between survivors (*n* = 60) and non-survivors (*n* = 19); all *χ*^2^ or Fisher exact *p* > 0.25 (Table 1). Survivors spent a median 33.5 (IQR 15.5–60) days in ICU versus 12 (IQR 4–21) days in ICU for non-survivors (*p* < 0.001). The Hodges–Lehmann estimate indicated a 20.5-day longer stay for survivors (95% CI + 11 to + 33) (Table 4).

**Table 4:** Univariable tests versus 1-year survival

Predictor	Test (*df*)	Test value	*p*-Value (two-sided)
Gender	*χ*^2^ (1)	1.06	0.303
Mechanism	*χ*^2^ (4)	3.62	0.486
Spinal region	*χ*^2^ (2)	0.25	0.882
GCS category	*χ*^2^ (2)	0.38	0.828
**LOS (days)**	**Mann–Whitney *z***	**-3.95**	** < 0.001**
Hodges–Lehmann shift	–	+ 20.5 days (95% CI + 11 to + 33)	–
Age ≥ 45 years	*χ*^2^ (1)	1.23	0.268
High-energy mechanism	*χ*^2^ (1)	0.31	0.580

High-energy mechanism = all except low energy and assault

LOS differed across discharge categories (Kruskal–Wallis *χ*^2^ = 10.03, *p* = 0.007): patients transferred to neurorehabilitation recorded the longest stays (median 52.5 days), whereas those discharged home stayed a median 18 days. LOS increased marginally (median 34 days) with thoracolumbar injuries (*p* = 0.051) than those with cervical (median 16 days) or combined injuries (median 24 days) but showed no relationship to GCS category (*p* = 0.607) (Table 3).

## Length of Stay by Discharge Destination, Spinal Column Region, and Admission GCS

To avoid potential confounding from early death due to severity of injury on the length of stay, we repeated the analyses, restricting all LOS analyses to survivors; only 65 adult patients with concomitant craniospinal trauma were analyzed (Supplementary Table 1).

**Fig. 3:**
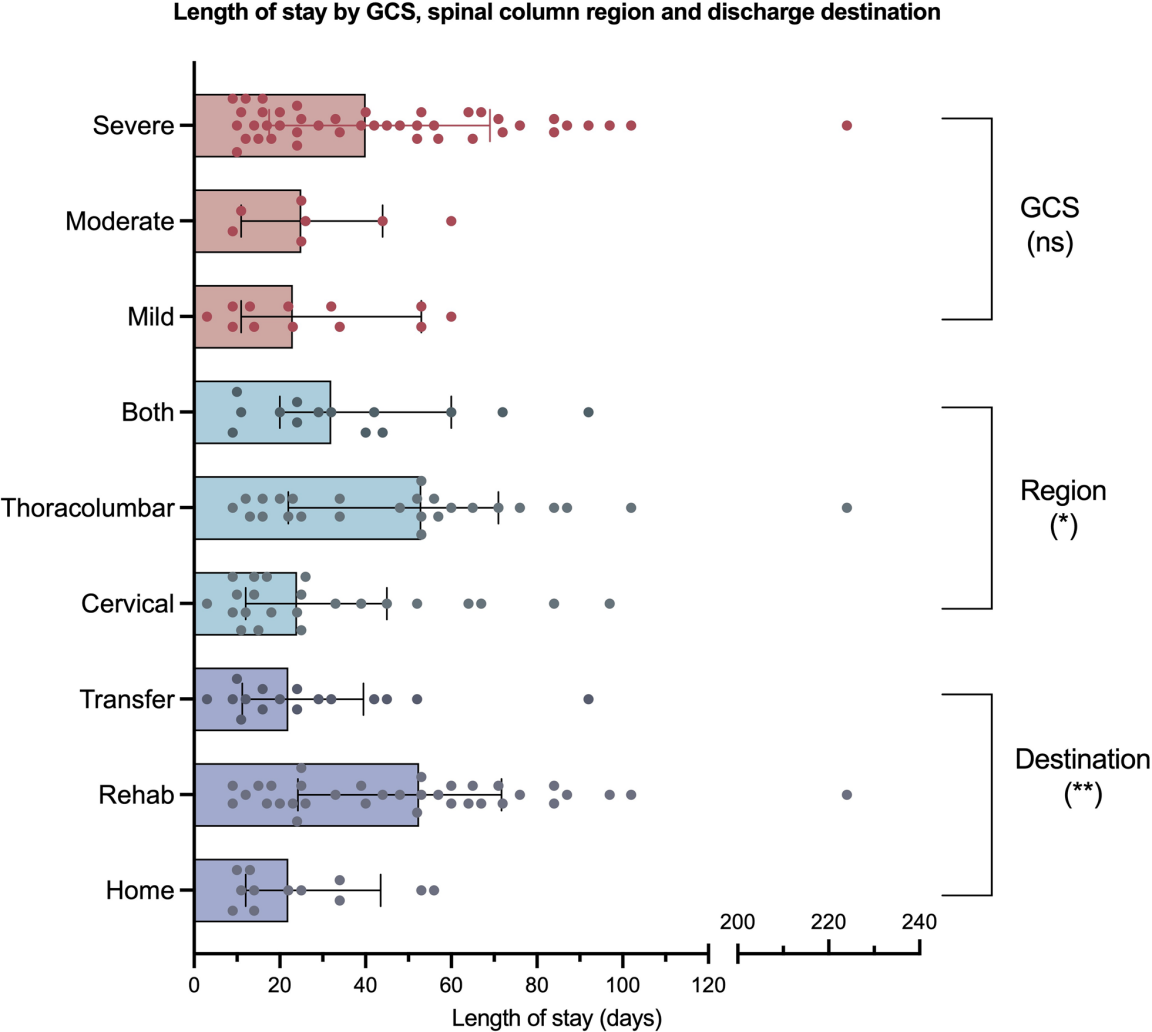
Length of stay by discharge destination, spinal region, and GCS category (in relation to Table 5)

Median age was 45 (IQR 29–54) years and 89.2% were male. Motor vehicle accidents (MVA) and high-energy falls together accounted for 84.6% of injuries, and thoracolumbar fractures predominated (41.5%). On admission, 69.2% presented with severe TBI (GCS ≤ 8) (Table 5 and Fig. 3).

- Discharge destination: LOS differed significantly (*p* < 0.01); rehabilitation had the longest stays [median 52.5 (24.5–71.5) days] vs. home [22 (13–34) days] and transfer [22 (11.5–37) days].
- Spinal region: borderline overall difference (*p* = 0.05); thoracolumbar had longer stays [median 53 (22–71) days] than cervical [24 (12–45) days]; “both” was intermediate [32 (20–60) days].
- GCS category: no evidence of difference (*p* = 0.21).

**Table 5:** LOS by discharge destination, spinal region, and severity (statistical significance denoted by * at < 0.05 and by ** at < 0.01) (see associated Fig. 3)

Grouping		Median [IQR] days	K-W *χ*^2^ (*df*)	*p*-Value
Discharge destination	Home (*n* = 12)	18 [12–43.5]	(2) = 10.03	0.007 **
	Rehabilitation (*n* = 34)	52.5 [24–72]		
	Transfer (*n* = 15)	24 [11–32]		
Spinal region	Thoracolumbar (*n* = 33)	34 [16–60]	(2) = 5.94	0.051*
	Cervical (*n* = 27)	16 [9–39]		
	Both (*n* = 19)	24 [11–44]		
GCS category	Mild (*n* = 16)	22.5 [10–43.5]	(2) = 0.99	0.607

For LOS, contrary to the a priori expectation of our hypothesis, the univariate and multivariable model showed lower odds of prolonged LOS with cervical injury. For discharge destination, unadjusted ORs for region and other predictors had wide CIs that crossed 1; there was no statistically significant association with home discharge.

## Predictors of Discharge Destination—Univariate Analysis

Age did not differ significantly between groups. Patients discharged home were a median of 6 years older compared with those not discharged home (HL estimate + 6 years, 95% CI − 6 to 20; *p* = 0.331).

**Fig. 4:**
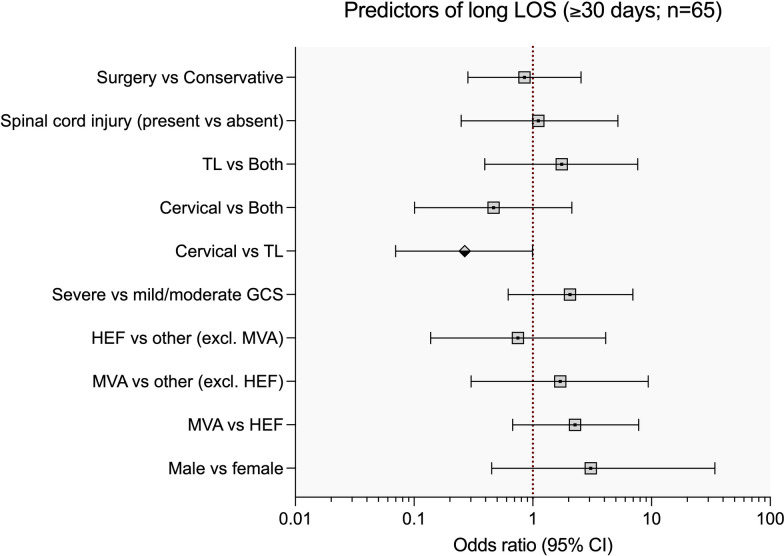
Predictors of long length of stay (in relation to Table 6)

Overall, there was no statistical significance that sex, spinal column injury mechanism, spinal column region, spinal cord injury, or operative management were associated with discharge home. Most odds ratios were imprecise, with wide confidence intervals crossing 1, reflecting small cell counts. Severe GCS (≤ 8) showed a trend toward lower odds of discharge home (OR 0.29, 95% CI 0.07–1.22; *p* = 0.09) (Fig. 4).

**Table 6:** Logistic regression: predictors of long LOS ≥ 30 days (see associated Fig. 4)

Predictors	OR	95% CI	*p*-Value
Age (per year increase)	1.02	0.99–1.05	0.299
Anatomical location of injury			
Cervical versus TL	0.26	0.08–0.86	0.027 *
Both versus TL	0.37	0.10–1.36	0.134
Injury mechanism			
Low-energy falls vs. MVA	0.27	0.02–3.33	0.306
High-energy falls vs. MVA	0.46	0.15–1.35	0.318
Bike versus MVA	1.74	0.21–14.35	0.606
GCS category			
Moderate versus mild	2.45	0.32–18.80	0.387
Severe versus mild	1.94	0.55–6.87	0.302

Model LR *χ*^2^(8) = 9.36, *p* = 0.313; pseudo *R*^2^ = 0.088

## Predictors of Prolonged Length of Stay (LOS ≥ 30 days): Univariate Analysis

**Fig. 5:**
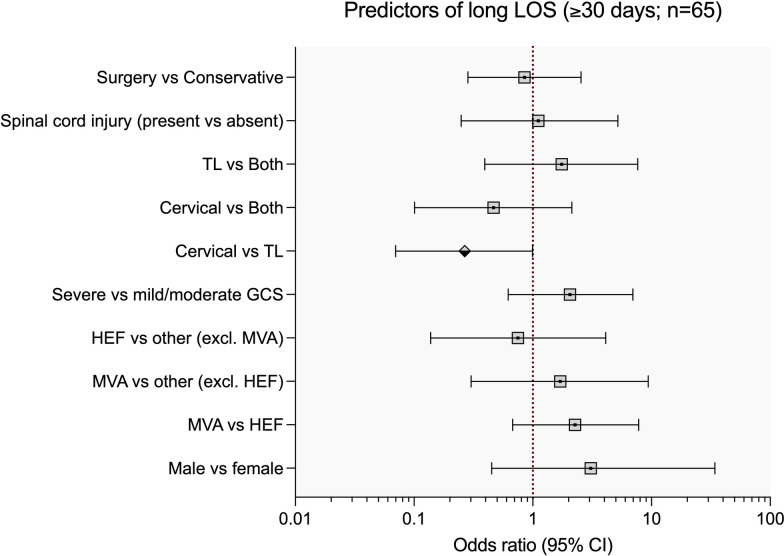
Favorable discharge (home) (in relation to Table 7)

LOS did not differ by age: patients with prolonged LOS were a median of 1 day younger than those with LOS < 30 days (HL estimate − 1 day, 95% CI − 10 to + 7; *p* = 0.87). Across sex, mechanism of spinal column injury, GCS, spinal cord injury, and management, there was no clear association with prolonged LOS (all *p* ≥ 0.18). The only comparison reaching significance was spinal column region: cervical vs. thoracolumbar injuries showed lower odds of prolonged LOS (OR 0.27, 95% CI 0.07–0.99; *p* = 0.03) (Fig. 5).

**Table 7:** Favorable discharge (home) logistic model (see associated Fig. 5)

Predictors	OR	95% CI	*p*-Value
Age (per year increase)	0.98	0.94–1.03	0.446
Severe versus mild GCS	0.12	0.03–0.49	0.003 **
*Anatomical location of injury*			
Cervical versus TL	0.88	0.19–4.00	0.864
Both versus TL	0.29	0.03–2.95	0.295

Model LR *χ*^2^ (4) = 11.34, *p* = 0.023; pseudo *R*^2^ = 0.176

## Adjusted Odds of Prolonged Length of Stay (LOS ≥ 30 days)—Multivariable Analysis

In the multivariable Firth’s logistic model (*n* = 65; outcome LOS ≥ 30 days), cervical (vs. non-cervical) spinal column injury was independently associated with lower odds of prolonged LOS (aOR 0.34, 95% CI 0.12–0.97, *p* = 0.04) after adjustment for age and GCS. Age (per 10 years) showed no association (aOR 1.02, 95% CI 0.75–1.37, *p* = 0.92). Severe GCS (≤ 8 vs. > 8) pointed toward higher odds but was not statistically significant (OR 2.07, 95% CI 0.70–6.07, *p* = 0.19).

## Adjusted Odds of Discharge Destination—Multivariable Analysis

**Fig. 6:**
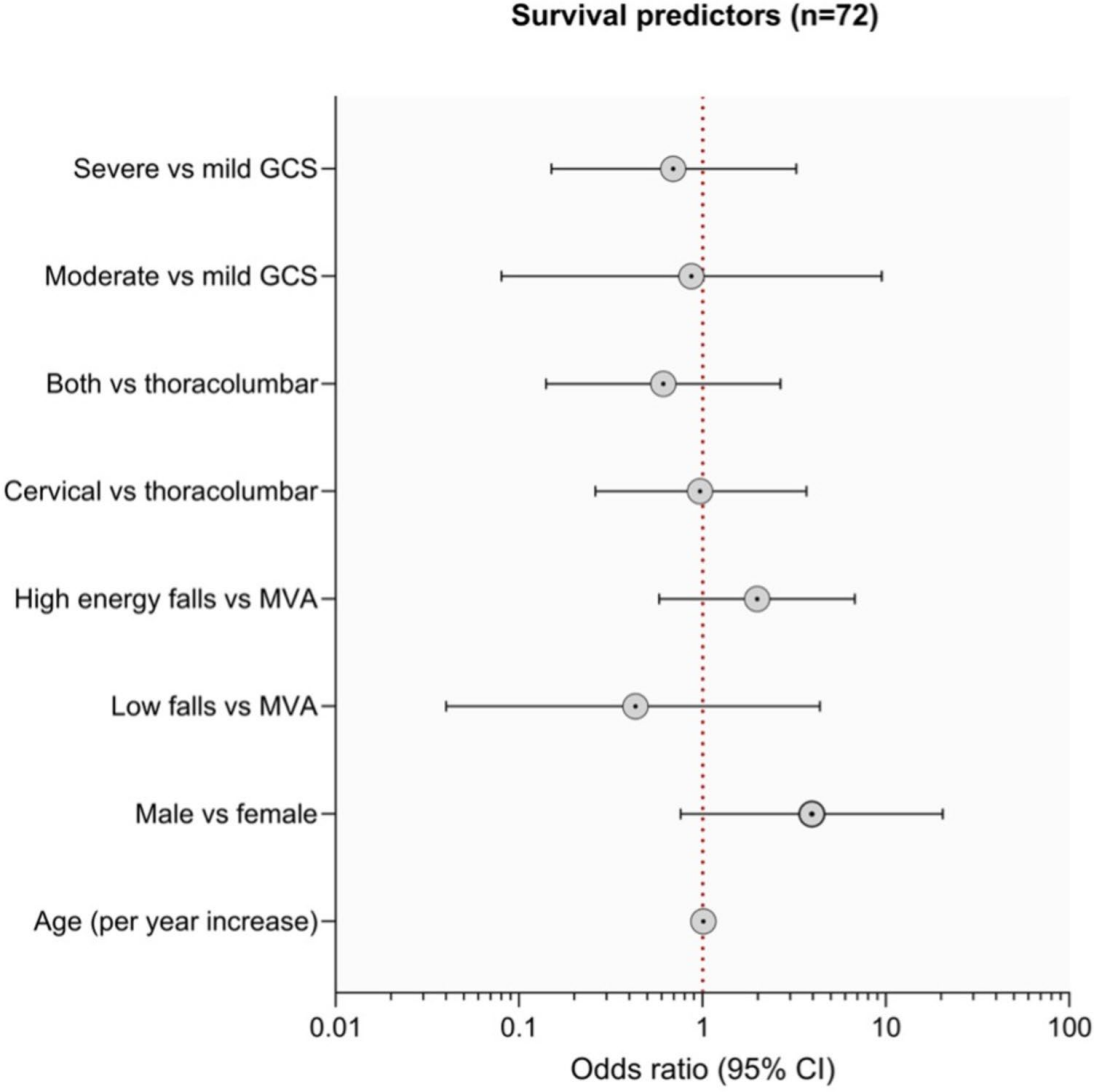
Predictors of survival (in relation to Table 8)

The number of home discharges was small (13/65), and several predictors produced sparse/zero cells (e.g., spinal cord injury had 0% discharged home). With our prespecified covariates (age, GCS, region, management), the events-per-variable (EPV) would fall well below conventional thresholds (≥ 10 EPV). Under these conditions a multivariable model would be unstable/overfitted, yielding inflated or extremely imprecise adjusted ORs. We therefore report unadjusted exact tests and ORs with exact CIs for discharge destination (Fig. 6).

**Table 8:** Logistical mode—Survival predictors (see associated Fig. 6)

Predictors	OR	95% CI	*p*-Value
Age (per year increase)	1.01	0.98–1.05	0.475
Male vs. female	3.94	0.76–20.41	0.103
*Injury mechanism*			
Low-energy falls versus MVA	0.43	0.04–4.36	0.476
High-energy falls versus MVA	1.98	0.58–6.76	0.274
*Anatomical location of injury*			
Cervical versus TL	0.97	0.26–3.68	0.964
Both versus TL	0.61	0.14–2.66	0.516
*GCS category*			
Moderate versus mild	0.87	0.08–9.50	0.912
Severe versus mild	0.69	0.15–3.24	0.642

Model LR *χ*^2^ (8) = 5.68, *p* = 0.683; pseudo *R*^2^ = 0.068

## Discussion

In our cohort of 560 patients admitted to ICU with TBI, the rate of concomitant spinal column injury was 15.2%, while the rate of a concomitant spinal cord injury was 2.9%. Risk factors for this group included male gender, age < 65 years, and a high-energy mechanism of injury. Unexpectedly, TBIs were associated with more thoracolumbar than cervical concomitant spinal trauma. Contrary to the a priori expectation of our hypothesis, the univariate and multivariable model showed lower odds of prolonged LOS with cervical injury. For discharge destination, unadjusted ORs for region and other predictors had wide CIs that crossed 1; there was no statistically significant association with home discharge. A high index of suspicion remains essential to identifying such associated patterns of injury. In-hospital mortality was 21.2% during index admission and 25.9% at 1 year.

## Patient Demographics and Associations

We found that more male than female individuals were admitted with TBI (441/560) and had an associated higher risk of sustaining a concomitant spinal injury; 16.5% of male compared with 10.1% of female individuals. Despite male individuals being at a higher risk, we found that female individuals were associated with poorer outcomes: 33.3% of female patients in our study died during admission compared with 19.2% of male patients. This is in contrast to some available studies, which reported that female individuals tend to have fewer complications and better prognosis than men after sustaining TBI or spinal injuries [23–25].

Endogenous hormones may influence sex-based differences in patient outcomes after TBI [21]. Estrogen is thought to have neuroprotective effects by reducing proinflammatory cytokines [27–29]. The mean age of female patients in our study was 47 years, with more than two-thirds of them being over the age of 40 years. With increasing age in the female population, the levels of estrogen decrease, putting post- and perimenopausal women at a higher risk of sustaining more severe TBI [30]. In comparison, the mean age of male patients in our study was 41 years, with just over half (53.4%) of them being over the age of 40. While testosterone also declines with age, its neuroprotective role remains unclear [31, 32]. We note that the small number of female patients in our study (*n* = 12) may limit the significance of our findings. Moving forward, larger and more balanced cohorts are required to effectively assess potential sex-based differences in patient outcomes.

When analyzing age, we found that 91.8% of patients admitted to our ICU were aged 65 years and under (78/85). Most epidemiological studies define the elderly population as the age group older than 65 years of age [33]. The disparity may reflect ICU triage practices, as elderly patients are at higher risk of mortality due to preexisting comorbidities, illness severity, and impaired consciousness, all of which are common in concomitant TBI and spinal trauma [34–36], which likely influenced ICU admission decisions. Although the small number of elderly patients limits statistical conclusions, our findings align with evidence that advanced age predicts poorer outcomes after TBI or spinal injury [37, 38].

## Mechanism of Injury and Associations

The vast majority (80/85; 94.1%) of patients who suffered concomitant TBI and spinal injury had sustained trauma with high-energy impact. One-fifth of these patients died during admission, indicating the high risk of poor outcomes when a dual neurotrauma injury pattern of both TBI and spinal injury occurs. This association has also been seen in other studies, even though those had focused on isolated injuries. For example, Carroll et al. showed that outcomes in patients such as quality of life and cognitive status were significantly poorer following severe TBI caused by high-energy impact mechanisms of injury [39]. Bak et al. showed that the sensorimotor function after traumatic spinal cord injury in patients with a high-energy mechanism of injury were associated with significantly lower motor recovery [40]. The substantial forces involved in these high-energy injuries can cause concomitant TBI and spinal injury due to the blunt impact and/or inertial loading onto the head/spine. Various factors such as the magnitude, direction, rotation, and timing of the forces sustained by these individuals need to be considered, especially during the secondary survey in assessing a patient with a major trauma [41]. This would allow clinicians to timely and accurately identify any risk of craniospinal injury and manage such injuries accordingly.

We also found that 58/80 patients with a high-energy impact mechanism of injury had severe TBI on admission, and 13/80 were found to have a spinal cord injury (SCI). In these patients with severe TBI, the reduced consciousness level does make it difficult to thoroughly assess the patients’ symptoms. Due to the nature and severity of the mechanism of injury sustained by these patients, it is important to note the necessity of a thorough secondary survey to identify and rule out any neurological deficits or injuries elsewhere. One study by Yi et al. showed that within a group of patients with TBI, they identified a subgroup with missed injuries including SCI, where 65% of them had a GCS score of less than 8. This was in comparison with another group with no missed injuries, where only 36% of patients had a GCS score of less than 8 [42]. In the context of high-energy impact injuries, it is imperative to always consider other injuries, and specifically the possibility of concomitant TBI and spinal trauma. The potential sequelae of overlooking other critical injuries that can affect patient outcomes can be overemphasized.

## Pattern of Spinal Column Injury

In our study, we found more concomitant thoracolumbar (34/85; 40%) than cervical spinal injuries (30/85; 35.3%). Nearly a quarter of patients with concomitant craniospinal trauma (21/85; 24.7%) had both cervical and thoracolumbar injuries.

Our findings were similar to some studies that showed that thoracolumbar spine injuries were more common alongside TBI in a traumatic setting [43, 44]. However, this contrasts with other studies, which showed that the cervical spine is most susceptible to injury based on its anatomy and flexibility [45, 46]. Dichotomizing between axial (C1/2) and subaxial (C3–7) regions, Fig. 2 showed fewer upper cervical spine injuries compared with subaxial. Also interesting is the observation that there was more trauma involving the proximal and distal junctional areas of the cervical spine than the mid-cervical region.

Our findings are comparable to other studies showing that lower cervical spine injuries from C3 to C7 are more common [12, 47]. The variability seen in the epidemiology of spinal injuries associated with TBI in different studies reinforces our opinion that more studies are needed to analyze the epidemiology of concomitant craniospinal trauma to further consolidate more information on this topic.

## Pattern of Spinal Cord Injury

Within our cohort of 85 patients with TBI and concomitant spinal column injury, nearly one-fifth of patients (18.8%; 16/85) had sustained a spinal cord injury (SCI). Unsurprisingly, 13/16 of these patients had sustained a high-energy impact mechanism of injury, while the remaining three had a low-energy impact.

One common characteristic in those three patients with low-energy impact was a background of chronic alcohol dependence. There is sparse information on the effects of chronic alcohol use being a risk factor for developing a SCI, but Garrison et al. showed that alcohol use at the time of a traumatic injury was associated with an increased risk of sustaining a cervical spinal cord injury [48]. More studies are required to study the relationship between alcohol use and the risk of sustaining a SCI.

Nearly a third of patients sustaining a concomitant SCI (5/16 patients; 31.3%) died during admission, indicating poorer outcomes in this subgroup. By comparison, the mortality rate in the subgroup of patients with concomitant column but not cord injury was 18.8% (13/69). In addition to the sensory and motor neurological dysfunction that occur, SCIs also have the potential to affect other body systems and cause cardiorespiratory compromise [49, 50], possibly contributing to the poorer outcomes seen in this subgroup of patients. Due to the high risk of morbidity and mortality, it is again imperative not to miss potential concomitant injuries to the spinal cord, and to have a high index of suspicion.

In a much larger studied population, very recent data from the Nationwide Trauma Registry in Japan showed that concomitant traumatic spinal cord injury (t-SCI) and TBI occurred in 3.2% of patients. The majority of concomitant injuries occurred with mild TBI, whereas with increasing severity of TBI, the less frequently t-SCI occurred. Interestingly, TBI-related deaths were predominantly found in severe TBI cases without concomitant t-SCI. The in-hospital mortality rates for t-SCI without TBI, TBI without t-SCI, and concomitant t-SCI + TBI were 2.6%, 10.8%, and 5.3%, respectively [50]. We agree with the conclusions of that study that there may exist a potential underestimation of t-SCI in severe TBI, and that this is an area that deserves increased attention.

## Patients Discharged to Neurorehabilitation Centers

In our study, we identified 36/85 (42.3%) patients who were transferred or referred to for further neurorehabilitation after their initial inpatient admission in our center. This was a relatively low percentage of patients who received neurorehabilitation, especially given the nature of the injuries sustained. Various studies have shown the positive effects that neurorehabilitation provides to patients, especially when commenced earlier. One study showed that implementing neurorehabilitation earlier in the process of recovery helped improve neurological and functional outcomes in patients with moderate-to-severe TBIs [51]. Moving forward, further consideration and thought needs to be made by clinicians to refer patients with concomitant injuries for neurorehabilitation, and to continue following up on them as an outpatient if deemed appropriate to monitor their recovery process.

It should also be noted that governmental bodies have a responsibility in the ongoing care of these patients after their initial injury. They can help by funding further research into new treatment options for both TBIs and spinal injuries. The emergence of research on this is encouraging, with some avenues, such as the effectiveness of stem cell therapy for spinal cord injury rehabilitation, being studied [52].

## Current Guidelines on Managing Both TBI and Spinal Injury

To the best of our knowledge, there are no available official guidelines from organizations or health bodies that specifically advise on the best approach to managing TBI with concomitant spinal injury. Our local guidelines, similar to elsewhere, are specific to individual TBI or traumatic spinal injury. They consider initial assessment, head and/or spine imaging, criteria for referral to neurosurgery, and ongoing management options on both these injuries individually [53, 54]. Internationally recognized guidelines, such as the Canadian C-Spine protocol, which has helped guide healthcare professionals in quantifying the risk and determining the need for any cervical spine imaging in trauma patients [55, 56], was designed mainly as a sensitive decision rule in determining cervical spine injuries in alert and stable patients with trauma, as it would require the patients to cooperate with a clinical examination.

With regard to the screening and evaluation for potential SCIs, the institutional guidelines depend on any positive neurological symptoms, or if CT of the spine had shown a suspicion of spinal cord damage or impingement. However, this would be difficult to utilize efficiently in patients with severe TBIs and with a consequentially reduced GCS score, as seen in the majority of patients in our study who had a GCS score of ≤ 8, and would not allow for a thorough neurological examination. Additionally, SCIs could also have happened without an associated spinal column injury. For example, these injuries can be seen in elderly patients with preexisting degenerative spinal changes, and central cord syndrome in patients with underlying spinal canal stenosis [57]. It is acknowledged that there is a theoretical possibility that potential SCIs may have been missed in certain individuals due to these possibilities. Such concerns reinforce the notion that protocols need to be designed to stratify risk and avoid missing potential SCIs. The recent emergence of guidelines for the management of SCI in the polytrauma setting is encouraging [58].

Lessons on the patterns of concomitant craniospinal injury and their outcomes can help stratify resources, improve the assessment and diagnosis of such complex trauma, and guide future protocols to improve patient outcomes.

## Limitations

This was a single-center retrospective study, which comes with several known constraints and limitations. One of these key limitations is that practice patterns in the southeast region of Scotland may not necessarily represent the broader demographics in parts of the United Kingdom or internationally. Additionally, the relatively small population has implications in detecting significant statistical associations or outcomes. This particularly applied to the small number of female patients in our study. Another limitation was that our study only managed to capture TBI cases that required admission to our ICU, and did not capture patients with mild-or-moderate TBI who were not deemed appropriate for transfer to the ICU. Spinal injuries with mild TBI were also possibly under-captured, as most of these cases would have been routed to the National Spinal Cord Injuries Centre in Glasgow, as per preexisting national referral pathways.

As is the case in retrospective studies, we relied on preexisting medical records, and there were instances of incomplete or undocumented information. Specifically, we were unable to follow up and obtain information on some patients who were subsequently transferred to different hospitals, due to different patient record systems used. This inevitably affected the data on 1-year outcomes in this subgroup of patients. We were also unable to obtain more specific information such as the functional status of patients at the time of discharge to better analyze patient outcomes. Moving forward, future research on TBI with concomitant spinal injuries could consolidate data from multiple study centers, allowing for a larger sample size and in turn producing results with a smaller margin of error.

Nevertheless, our study was pragmatic and longitudinally collected data over a period of 12 years, which is therefore one of the longest periods of such specific observation, providing useful epidemiological information.

## Conclusions

While there are numerous studies focusing on the epidemiology of TBI and spinal injury individually, dual neurotrauma injury patterns remain under-studied. Our study, despite its limitations, provides new insight into the epidemiology of concurrent brain and spinal neurotrauma.

The rate of concomitant spinal column injury was 15.2%, while the rate of concomitant spinal cord injury was 2.9%, in a group of 560 patients admitted with TBI to the southeast Scotland tertiary neurosurgery center in Edinburgh. Risk factors for this group included male gender, age 65 and younger, and a high-energy mechanism of injury. TBI was associated with more thoracolumbar than cervical concomitant spinal trauma. Length of stay in hospital was statistically significantly associated with discharge destination, region of spinal injury (longer stay with thoracolumbar injury), and 1-year survival.

A high index of suspicion remains essential to prevent missing such associated patterns of injury. In-hospital mortality was 21.2% during index admission and 25.9% at 1 year.

To consolidate information, further understand pathophysiology, and help guide specialized management of dual neurotrauma injuries, a concerted effort will be required both within and across healthcare systems with the ultimate aim of facilitating better outcomes in this special cohort of patients.

## Supplementary Information

Below is the link to the electronic supplementary material.

Supplementary file1 (DOCX 15 kb)

## References

[1] Capizzi A, Woo J, Verduzco-Gutierrez M. Traumatic brain injury: an overview of epidemiology, pathophysiology, and medical management. Med Clin North Am. 2020;104(2):213–38. 10.1016/j.mcna.2019.11.001. 32035565 10.1016/j.mcna.2019.11.001

[2] Zhang S, Wadhwa R, Haydel J, Toms J, Johnson K, Guthikonda B. Spine and spinal cord trauma: diagnosis and management. Neurol Clin. 2013;31(1):183–206. 10.1016/j.ncl.2012.09.012. 23186900 10.1016/j.ncl.2012.09.012

[3] Anjum A, Yazid MD, Fauzi Daud M, Idris J, Ng AMH, Selvi Naicker A, et al. Spinal cord injury: pathophysiology, multimolecular interactions, and underlying recovery mechanisms. Int J Mol Sci. 2020;21(20):7533. 10.3390/ijms21207533. 33066029 10.3390/ijms21207533 PMC7589539

[4] Thapa K, Khan H, Singh TG, Kaur A. Traumatic brain injury: mechanistic insight on pathophysiology and potential therapeutic targets. J Mol Neurosci : MN. 2021;71(9):1725–42. 10.1007/s12031-021-01841-7. 33956297 10.1007/s12031-021-01841-7

[5] Swartz EE, Floyd RT, Cendoma M. Cervical spine functional anatomy and the biomechanics of injury due to compressive loading. J Athl Train. 2005;40(3):155–61. 16284634 PMC1250253

[6] Nightingale RW, McElhaney JH, Richardson WJ, Myers BS. Dynamic responses of the head and cervical spine to axial impact loading. J Biomech. 1996;29(3):307–18. 10.1016/0021-9290(95)00056-9. 8850637 10.1016/0021-9290(95)00056-9

[7] Robba C, Bonatti G, Pelosi P, Citerio G. Extracranial complications after traumatic brain injury: targeting the brain and the body. Curr Opin Crit Care. 2020;26(2):137–46. 10.1097/MCC.0000000000000707. 32004191 10.1097/MCC.0000000000000707

[8] GBD 2016 Traumatic Brain Injury and Spinal Cord Injury Collaborators. Global, regional, and national burden of traumatic brain injury and spinal cord injury, 1990-2016: a systematic analysis for the Global Burden of Disease Study 2016. The Lancet. Neurology. 2019;18(1): 56–87. 10.1016/S1474-4422(18)30415-0 10.1016/S1474-4422(18)30415-0 PMC6291456 30497965

[9] Hyder AA, Wunderlich CA, Puvanachandra P, Gururaj G, Kobusingye OC. The impact of traumatic brain injuries: a global perspective. NeuroRehabil. 2007;22(5):341–53. 18162698

[10] Kumar R, Lim J, Mekary RA, Rattani A, Dewan MC, Sharif SY, et al. Traumatic spinal injury: global epidemiology and worldwide volume. World Neurosurg. 2018;113:e345–63. 10.1016/j.wneu.2018.02.033. 29454115 10.1016/j.wneu.2018.02.033

[11] Pandrich MJ, Demetriades AK. Prevalence of concomitant traumatic cranio-spinal injury: a systematic review and meta-analysis. Neurosurg Rev. 2020;43(1):69–77. 10.1007/s10143-018-0988-3. 29882173 10.1007/s10143-018-0988-3 PMC7010651

[12] Marchesini N, Demetriades AK, Peul, et al. Concomitant trauma of brain and upper cervical spine: lessons in injury patterns and outcomes. European J Trauma Emergency Surg. 2023. 10.1007/s00068-023-02278-w. 10.1007/s00068-023-02278-w PMC11599623 37184568

[13] Dion PM, Lapierre M, Said H, Tremblay S, Tariq K, Lamb T, et al. Rethinking cervical spine clearance in obtunded trauma patients: an updated systematic review and meta-analysis. Injury. 2024;55(3):111308. 10.1016/j.injury.2023.111308. 38266326 10.1016/j.injury.2023.111308

[14] Oner C, Rajasekaran S, Chapman JR, Fehlings MG, Vaccaro AR, Schroeder GD, et al. Spine trauma-What are the current controversies? J Orthop Trauma. 2017;31(Suppl 4):S1–6. 10.1097/BOT.0000000000000950. (**PMID: 28816869**). 10.1097/BOT.0000000000000950 28816869

[15] Riemann L, Alhalabi OT, Unterberg AW, Younsi A, CENTER-TBI investigators and participants. Concomitant spine trauma in patients with traumatic brain injury: patient characteristics and outcomes. Front Neurol. 2022;13:861688. 10.3389/fneur.2022.861688. 36062004 10.3389/fneur.2022.861688 PMC9436444

[16] Vattipally VN, Aude CA, Ran KR, Jiang K, Ranganathan S, Weber-Levine C, et al. Association between concomitant traumatic brain injury and unfavorable 1-year outcomes in patients with traumatic spinal cord injury. J Neurosurg Spine. 2025;43(3):375–83. 10.3171/2025.3.SPINE241470. (**PMID: 40577852**). 40577852 10.3171/2025.3.SPINE241470

[17] Azad TD, Raj D, Ran KR, Vattipally VN, Warman A, Raad M, et al. Concomitant traumatic brain injury delays surgery in patients with traumatic spinal cord injury. Neurosurgery. 2024. 10.1227/neu.0000000000002816. 10.1227/neu.0000000000002816 38197654

[18] Demetriades AK. Traumatic Brain Injury in Adults Service Mapping Report. Scottish Acquired Brain Injury Network (SABIN). 2020. https://www.sabin.scot.nhs.uk/wp-content/uploads/sites/10/2020/07/Service-Mapping-Report-Traumatic-Brain-Injury-in-Adults.pdf

[19] NHS Scotland. Scottish Intensive Care Society Audit Group. 2023. https://www.sicsag.scot.nhs.uk/about/main.html

[20] InterSystems. TrakCare. 2024. https://www.intersystems.com/uk/trakcare/

[21] NHS Scotland. Queen Elizabeth National Spinal Injuries Unit. 2024. https://www.spinalunit.scot.nhs.uk/

[22] NHS Lothian. Astley Ainslie Hospital. 2023. https://www.nhslothian.scot/goingtohospital/astley-ainslie-hospital/

[23] Groswasser Z, Cohen M, Keren O. Female TBI patients recover better than males. Brain Inj. 1998;12(9):805–8. 10.1080/026990598122197. 9755371 10.1080/026990598122197

[24] Saban KL, Smith BM, Collins EG, Pape TL. Sex differences in perceived life satisfaction and functional status one year after severe traumatic brain injury. J Womens Health (Larchmt). 2011;20(2):179–86. 10.1089/jwh.2010.2334. 21314444 10.1089/jwh.2010.2334

[25] Stewart AN, MacLean SM, Stromberg AJ, Whelan JP, Bailey WM, Gensel JC, et al. Considerations for studying sex as a biological variable in spinal cord injury. Front Neurol. 2020;11:802. 10.3389/fneur.2020.00802. 32849242 10.3389/fneur.2020.00802 PMC7419700

[26] Mollayeva T, Mollayeva S, Colantonio A. Traumatic brain injury: sex, gender and intersecting vulnerabilities. Nat Rev Neurol. 2018;14(12):711–22. 10.1038/s41582-018-0091-y. 30397256 10.1038/s41582-018-0091-y

[27] Lawrence CB, Allan SM, Rothwell NJ. Interleukin-1beta and the interleukin-1 receptor antagonist act in the striatum to modify excitotoxic brain damage in the rat. Eur J Neurosci. 1998;10(3):1188–95. 10.1046/j.1460-9568.1998.00136.x. 9753187 10.1046/j.1460-9568.1998.00136.x

[28] Olsson T. Critical influences of the cytokine orchestration on the outcome of myelin antigen-specific T-cell autoimmunity in experimental autoimmune encephalomyelitis and multiple sclerosis. Immunol Rev. 1995;144:245–68. 10.1111/j.1600-065x.1995.tb00072.x. 7590816 10.1111/j.1600-065x.1995.tb00072.x

[29] Khaksari M, Abbasloo E, Dehghan F, Soltani Z, Asadikaram G. The brain cytokine levels are modulated by estrogen following traumatic brain injury: which estrogen receptor serves as modulator? Int Immunopharmacol. 2015;28(1):279–87. 10.1016/j.intimp.2015.05.046. 26112336 10.1016/j.intimp.2015.05.046

[30] Blaya MO, Raval AP, Bramlett HM. Traumatic brain injury in women across lifespan. Neurobiol Dis. 2022;164:105613. 10.1016/j.nbd.2022.105613. 34995753 10.1016/j.nbd.2022.105613

[31] Cheng H, Zhang X, Li Y, et al. Age-related testosterone decline: mechanisms and intervention strategies. Reprod Biol Endocrinol. 2024;22(1):144. 10.1186/s12958-024-01316-5. 39543598 10.1186/s12958-024-01316-5 PMC11562514

[32] Coyoy-Salgado A, Segura-Uribe J, Salgado-Ceballos, et al. Evaluating sex steroid hormone neuroprotection in spinal cord injury in animal models: is it promising in the clinic? Biomedicines. 2024;12(7):1478. 39062051 10.3390/biomedicines12071478 PMC11274729

[33] Ferrucci L, Giallauria F, Guralnik JM. Epidemiology of aging. Radiol Clin North Am. 2008;46(4):643–v. 10.1016/j.rcl.2008.07.005. 18922285 10.1016/j.rcl.2008.07.005 PMC2692491

[34] Chelluri L, Im KA, Belle SH, et al. Long-term mortality and quality of life after prolonged mechanical ventilation. Crit Care Med. 2004;32:61–9. 14707560 10.1097/01.CCM.0000098029.65347.F9

[35] Jandziol AK, Ridley SA. Validation of outcome prediction in elderly patients. Anaesthesia. 2000;55:107–12. 10651669 10.1046/j.1365-2044.2000.055002107.x

[36] Vosylius S, Sipylaite J, Ivaskevicius J. Determinants of outcome in elderly patients admitted to the intensive care unit. Age Ageing. 2005;34(2):157–62. 10.1093/ageing/afi037. 15713860 10.1093/ageing/afi037

[37] Gardner RC, Dams-O’Connor K, Morrissey MR, Manley GT. Geriatric traumatic brain injury: epidemiology, outcomes, knowledge gaps, and future directions. J Neurotrauma. 2018;35(7):889–906. 10.1089/neu.2017.5371. 29212411 10.1089/neu.2017.5371 PMC5865621

[38] Furlan JC, Fehlings MG. The impact of age on mortality, impairment, and disability among adults with acute traumatic spinal cord injury. J Neurotrauma. 2009;26(10):1707–17. 10.1089/neu.2009.0888. 19413491 10.1089/neu.2009.0888 PMC2822797

[39] Carroll EL, Manktelow AE, Outtrim JG, Chatfield D, Forsyth F, Hutchinson PJA, et al. Influence of concomitant extracranial injury on functional and cognitive recovery from mild versus moderate-to- severe traumatic brain injury. J Head Trauma Rehabil. 2020;35(6):E513–23. 10.1097/HTR.0000000000000575. 32472833 10.1097/HTR.0000000000000575

[40] Bak AB, Moghaddamjou A, Malvea A, Fehlings MG. Impact of mechanism of injury on long-term neurological outcomes of cervical sensorimotor complete acute traumatic spinal cord injury. Neurospine. 2022;19(4):1049–56. 10.14245/ns.2244518.259. 36597641 10.14245/ns.2244518.259 PMC9816602

[41] Thompson, W. L., Stiell, I. G., Clement, C. M., Brison, R. J., Canadian C-Spine Rule Study Group . Association of injury mechanism with the risk of cervical spine fractures. CJEM. 2009;11(1) 14–22. 10.1017/s1481803500010873 10.1017/s1481803500010873 19166635

[42] Yi F, Zhonghua S, Yuhai W, Jirong D, Xuejian C. Missed injury in patients with severe traumatic brain injury complicated by multiple trauma. Turk Neurosurg. 2013;23(2):198–201. 10.5137/1019-5149.JTN.6669-12.1. 23546905 10.5137/1019-5149.JTN.6669-12.1

[43] Häske D, Lefering R, Stock JP, Kreinest M, TraumaRegister DGU. Epidemiology and predictors of traumatic spine injury in severely injured patients: implications for emergency procedures. European J Trauma Emergency Surg: Official Publ European Trauma Soc. 2022;48(3):1975–83. 10.1007/s00068-020-01515-w. 10.1007/s00068-020-01515-w PMC9192373 33025171

[44] Laurer H, Maier B, El Saman A, Lehnert M, Wyen H, Marzi I. Distribution of spinal and associated injuries in multiple trauma patients. Eur J Trauma Emerg Surg. 2007;33(5):476–81. 10.1007/s00068-007-7124-3. 26814932 10.1007/s00068-007-7124-3

[45] Torlincasi AM, Waseem M. Cervical Injury. In StatPearls: StatPearls Publishing; 2022. 28846253

[46] van Den Hauwe L, Sundgren PC, Flanders AE. Spinal Trauma and Spinal Cord Injury (SCI). Diseases of the Brain, Head and Neck, Spine 2020–2023: Diagnostic Imaging [Internet]. Cham (CH): Springer; 2020. https://www.ncbi.nlm.nih.gov/books/NBK554330/32119240

[47] Bennett J, Das JM, Emmady PD. Spinal Cord Injuries. StatPearls. Treasure Island (FL): StatPearls Publishing;2024. https://www.ncbi.nlm.nih.gov/books/NBK560721/

[48] Garrison A, Clifford K, Gleason SF, Tun CG, Brown R, Garshick E. Alcohol use associated with cervical spinal cord injury. J Spinal Cord Med. 2004;27(2):111–5. 10.1080/10790268.2004.11753740. 15162880 10.1080/10790268.2004.11753740 PMC1350951

[49] Dave S, Dahlstrom JJ, Weisbrod LJ. Neurogenic Shock. In StatPearls: StatPearls Publishing; 2023. 29083597

[50] Tachino J, Demetriades AK, Peul W, et al. Effects of concomitant traumatic spinal cord and brain injury on in-hospital mortality: a retrospective analysis of a nationwide trauma registry in Japan. J Neurotrauma. 2024. 10.1089/neu.2024.0168. 10.1089/neu.2024.0168 38877809

[51] Königs M, Beurskens EA, Snoep L, Scherder EJ, et al. Effects of timing and intensity of neurorehabilitation on functional outcome after traumatic brain injury: a systematic review and meta-analysis. Arch Phys Med Rehabil. 2018. 10.1016/j.apmr.2018.01.013. 10.1016/j.apmr.2018.01.013 29428344

[52] Zipser CM, Cragg JJ, Guest JD, Fehlings MG, Jutzeler CR, Anderson AJ, et al. Cell-based and stem-cell-based treatments for spinal cord injury: evidence from clinical trials. Lancet Neurol. 2022;21(7):659–70. 10.1016/S1474-4422(21)00464-6. 35569486 10.1016/S1474-4422(21)00464-6

[53] NHS Lothian. Traumatic brain injury. South East Scotland Major Trauma Guidelines. Right Decisions for Health and Care. 2021. https://rightdecisions.scot.nhs.uk/south-east-scotland-major-trauma-guidelines/neurosurgerymaxillofacial/traumatic-brain-injury/

[54] NHS Lothian. Spinal column & spinal cord injuries. South East Scotland Major Trauma Guidelines. Right Decisions for Health and Care. 2021. https://rightdecisions.scot.nhs.uk/south-east-scotland-major-trauma-guidelines/neurosurgerymaxillofacial/spinal-column-spinal-cord-injuries/

[55] Stiell IG, Wells GA, Vandemheen KL, et al. The Canadian C-spine rule for radiography in alert and stable trauma patients. JAMA. 2001;286(15):1841–8. 10.1001/jama.286.15.1841. 11597285 10.1001/jama.286.15.1841

[56] Baratloo A, Ahmadzadeh K, Forouzanfar MM, et al. NEXUS vs. Canadian C-spine rule (CCR) in predicting cervical spine injuries; a systematic review and meta-analysis. Arch Acad Emerg Med. 2023;11(1):e66. 10.22037/aaem.v11i1.2143. 37840870 10.22037/aaem.v11i1.2143 PMC10568954

[57] Koyanagi I, Iwasaki Y, Hida K, et al. Acute cervical cord injury without fracture or dislocation of the spinal column. J Neurosurg. 2000;93(1 Suppl):15–20. 10.3171/spi.2000.93.1.0015. 10879753 10.3171/spi.2000.93.1.0015

[58] Picetti E, Demetriades AK, Catena F, et al. Early management of adult traumatic spinal cord injury in patients with polytrauma: a consensus and clinical recommendations jointly developed by the World Society of Emergency Surgery (WSES) & the European Association of Neurosurgical Societies (EANS). World J Emerg Surg. 2024;19(1):4. 10.1186/s13017-023-00525-4. 38238783 10.1186/s13017-023-00525-4 PMC10795357

