# Supplementary material for: The Burden of Concomitant Spinal Injury in the Setting of Traumatic Brain Injury that Required Admission to ICU—Lessons from a Tertiary Neurosurgery Center
Source: Neurocrit Care. 2026 Feb 18;44(3):922–38. doi: 10.1007/s12028-026-02454-x (PMC13249621; doi:10.1007/s12028-026-02454-x)
Supplement: Supplementary file 1 — Supplementary file1 (DOCX 15 kb) [file 12028_2026_2454_MOESM1_ESM.docx]

**Supplementary Table 1. Baseline characteristics of survivors (n=65).**

| **Variable** | | **Survived n=65 (%)** |
| --- | --- | --- |
| **Age, years** | *Mean± SD* | *42.8±16.4* |
| **Sex** | Female | 7 (10.8) |
|  | Male | 58 (89.2) |
| **Mechanism** | MVA | 27 (41.5) |
|  | Low energy | 3 (4.6) |
|  | High-energy fall | 28 (43.1) |
|  | Bicycle | 6 (9.2) |
|  | Assault | 1 (1.5) |
| **Spinal column injury** | Thoraco-lumbar | 27 (41.5) |
|  | Cervical | 23 (35.4) |
|  | Both | 15 (23.1) |
| **Spinal cord injury** | Present | 11 (16.9) |
|  | Not present | 54 (83.1) |
| **Admission GCS** | Mild (13-15) | 13 (20.0) |
|  | Moderate (9-12) | 7 (10.8) |
|  | Severe (≤8) | 45 (69.2) |
| **Management** | Conservative | 37 (56.9) |
|  | Surgical | 28 (43.1) |
| **LOS, days** | *Median [IQR]* | *33.0 [28.5-55]* |
| **Discharge** | Home | 13 (20.0) |
|  | Rehab | 36 (55.4) |
|  | Transfer | 16 (24.6) |
